# Avoidance of Total Knee Arthroplasty in Early Osteoarthritis of the Knee with Intra-Articular Implantation of Autologous Activated Peripheral Blood Stem Cells versus Hyaluronic Acid: A Randomized Controlled Trial with Differential Effects of Growth Factor Addition

**DOI:** 10.1155/2017/8925132

**Published:** 2017-09-19

**Authors:** Thana Turajane, Ukrit Chaveewanakorn, Warachaya Fongsarun, Jongjate Aojanepong, Konstantinos I. Papadopoulos

**Affiliations:** ^1^Department of Orthopedic Surgery, Police General Hospital, Bangkok, Thailand; ^2^THAI StemLife Co. Ltd., Bangkok, Thailand; ^3^Department of Gynecology and Obstetrics, Police General Hospital, Bangkok, Thailand

## Abstract

In this randomized controlled trial, in early osteoarthritis (OA) that failed conservative intervention, the need for total knee arthroplasty (TKA) and WOMAC scores were evaluated, following a combination of arthroscopic microdrilling mesenchymal cell stimulation (MCS) and repeated intra-articular (IA) autologous activated peripheral blood stem cells (AAPBSCs) with growth factor addition (GFA) and hyaluronic acid (HA) versus IA-HA alone. Leukapheresis-harvested AAPBSCs were administered as three weekly IA injections combined with HA and GFA (platelet-rich plasma [PRP] and granulocyte colony-stimulating factor [hG-CSF]) and MCS in group 1 and in group 2 but without hG-CSF while group 3 received IA-HA alone. Each group of 20 patients was evaluated at baseline and at 1, 6, and, 12 months. At 12 months, all patients in the AAPBSC groups were surgical intervention free compared to three patients needing TKA in group 3 (*p* < 0.033). Total WOMAC scores showed statistically significant improvements at 6 and 12 months for the AAPBSC groups versus controls. There were no notable adverse events. We have shown avoidance of TKA in the AAPBSC groups at 12 months and potent, early, and sustained symptom alleviation through GFA versus HA alone. Differential effects of hG-CSF were noted with an earlier onset of symptom alleviation throughout.

## 1. Introduction

Osteoarthritis (OA) of the knee due to degenerative changes of the articular hyaline cartilage along with subchondral sclerosis, osteophyte formation, and synovitis represents a clinical conundrum that, due to its increasing prevalence in aging societies, places increasing burden on societal, financial, personal, and medical levels [1]. Articular hyaline cartilage is a specialized, low-friction, and wear-resistant surface tissue in weight-bearing diarthrodial joints with its main function to absorb, cushion, and protect the underlying bone from forces generated while the joint is being used. Its avascular, alymphatic nature complicates and often gravely impedes its capacity for regeneration and self-healing [2]. Trauma or osteoarthritis (OA) creates full-thickness chondral defects that cannot efficiently heal, thus leading to significant long-term disability [1]. Conventional treatments are unable to reverse or halt OA progression and are thus often restricted to activity modification, weight loss, and the management of symptoms (pain and inflammation) that, depending on disease severity, range from conservative treatment to surgical intervention, including total knee arthroplasty (TKA), with the numbers and costs of the latter having doubled between 2001 and 2012 [3]. Projections for 2030 estimate a 7-fold increase in TKA, while 67 million Americans will be afflicted by OA [4]. Chronic conventional treatments with anti-inflammatory agents, especially in seniors, are marred by gastrointestinal and renal side effects while surgical interventions may be complicated by deep-vein thromboses, joint stiffness, and muscle atrophy and importantly by prosthesis failure due to infections, wear, and loosening especially in younger age groups [3, 5, 6]. Moreover, many senior patients may not be ideal candidates for surgery due to cardiovascular conditions [4] while in younger age groups, a higher risk of revision, especially for aseptic failure, is evident [6]. Current knowledge indicates that in patients who are younger than 55 years, TKA should only be used in selected cases when there are no other satisfactory means of giving relief from pain and dysfunction [6]. Importantly, none of the above interventional therapeutic modalities address the need for hyaline cartilage regeneration. Arthroscopic debridement/microdrilling alone is not recommended for OA since there appear to be no benefits compared to a sham operation [7]. Patients treated with intra-articular hyaluronic acid injections (IA-HA) show clear symptomatic improvement and the intervention appears cost effective in up to 2 years of follow-up [8], but whether cartilage regeneration actually occurs is open to debate [9]. Neither autologous chondrocyte implantation nor microdrilling alone seems to result in cartilage resurfacing histologically resembling normal articular cartilage [10].

In view of OA being the leading cause of disability in the US, due to its increasing prevalence in our rapidly aging societies, there is a dire and acute need for a treatment modality that can demonstrate efficacy in preventing the progression of this degenerative joint disease, reverse its course, and offer cartilage regeneration options [11]. Cellular therapy for human adult use usually means that autologous nucleated cells are harvested via either bone marrow (BM) aspiration or peripheral blood (PB) stem cell collection through leukapheresis or via liposuction [11]. Cellular therapies for treating various stages of human OA have recently provided successful and encouraging clinical and laboratory results using the combination of intra-articular (IA) autologous activated peripheral blood stem cells (AAPBSCs) [12–16] with or without growth factor addition (GFA) [17, 18] along with hyaluronic acid (HA) in conjunction with arthroscopic microdrilling mesenchymal cell stimulation (MCS) in regenerating articular cartilage. In this randomized controlled trial, in early OA that failed conservative intervention, the clinical outcomes [primary endpoint: the need for total knee arthroplasty (TKA); secondary endpoint: WOMAC scores] following a combination of arthroscopic microdrilling mesenchymal cell stimulation (MCS) and repeated intra-articular (IA) autologous activated peripheral blood stem cells (AAPBSCs) with and without growth factor addition (GFA) along with hyaluronic acid (HA) versus IA-HA alone were evaluated.

## 2. Methods

### 2.1. Participants

Between March 1, 2011, and April 30, 2013, sixty patients (41 females) were recruited at the Police General Hospital's Orthopedic Outpatient Department for the present study and were randomized to one of three treatment groups, each consisting of twenty patients. The diagnosis of osteoarthritis (OA) was made by clinical, radiological, and arthroscopic evaluation. Chondral lesions were graded according to the International Cartilage Repair Society (ICRS) Cartilage Injury Evaluation Package [19]. The inclusion criteria were OA of the knee classified as Kellgren-Lawrence (KL) stages 1–3, ICRS grade III and IV lesions, failed conservative treatment for more than six months, not more than 3-degree varus or valgus deformity, and visual analog scale (VAS) scores of more than 40 in patients below the age of 60 years. Patients were excluded if they were older than 60 years and had secondary osteoarthritis, plica, intra-articular loose bodies, inflammatory joint disease, intra-articular steroid injection within the last six months, intra-articular hyaluronic acid within the last three months, prior glucosamine sulphate treatment, varus deformity more than 3 degrees, severe osteoarthritis (Kellgren-Lawrence stage > 4), known systemic or metabolic disease, malignancy, allergy to hyaluronic acid, allergy or known prior reaction to human granulocyte colony-stimulating factor (hG-CSF), severe or uncontrolled diabetes and/or proliferative diabetic retinopathy, obesity with BMI greater than 30, and prior stem cell treatment. All patients have been followed up for a minimum average of 12 months.

Patient demographics are shown in Table 1. Thirty-three of the 60 patients were in Kellgren-Lawrence grade 2, while the remainder were in grade 3, equally distributed in all 3 groups (Table 2). ICRS grade 3 was seen in 12 and 10 patients in groups 1 and 2, respectively, while 10 of each were ICRS grade 4. All patients had previously received conventional nonsteroidal anti-inflammatory drugs (NSAIDs), but none had received intra-articular corticosteroid injections. The study protocol was approved by the hospital's Ethics Committee and the National Medical Council. All patients signed informed consent forms after having received adequate information on the peripheral blood stem cell collection and discussed the procedure with the treating surgeon.

The recruited patients (*N* = 60: 41 females/19 males) were randomized to one of the three treatment groups, each consisting of twenty patients. The patients were randomized on a “first in study” basis using a 2-to-1 rule, meaning that 2 consecutive patients were randomized to the active groups 1 and 2, respectively, while the 3rd consecutive one was randomized to be a control in group 3 until 60 patients were enrolled. Those 2 consecutive patients knew that stem cells would be collected and administered but did not know which combination would be given, and neither did the treating doctor. Patients in group 1 (10 F/10 M) received the combination of IA AAPBSC with GFA consisting of platelet-rich plasma (PRP) and hG-CSF along with HA (2 ml Ostenil®, MW = 1.2 Mdal) in conjunction with arthroscopic MCS while group 2 (17 F/3 M) received the same combination with the exception of hG-CSF. Patients in group 3 (14 F/6 M) only received IA-HA without any arthroscopic surgery. Treatment between the groups that received stem cells (groups 1 and 2) and group 3 was open but blinded between the 2 stem cell groups (group 1 versus group 2). Each group received one weekly injection for 3 consecutive weeks and was evaluated at 0, 1, 6, and 12 months, with the primary endpoint and secondary endpoint being avoidance of TKA intervention and WOMAC scores, respectively.

### 2.2. Procedure Outline

The protocol comprised three steps: at first, AAPBSCs were harvested by a single hematologist (WF) and by leukapheresis in a Cobe Spectra apheresis machine (Caridian BCT, Denver, CO) following a 5-day stimulation with subcutaneous injections of hG-CSF at a dose of 5 *μ*g/kg BW/day. Following the harvest, PBSCs were tested for sterility (bacteria, viruses, and fungi), and after a fresh aliquot of 3 ml was reserved, part of the remaining portion was cryogenically frozen in 10% dimethyl sulphoxide and preserved in two cryovials of 4.5 ml to be thawed at the desired time of the future intra-articular injections. Pre- and postthaw measurements of viability (Table 3) and total nucleated cells (TNC) and CD34^+^ indicating hematopoietic stem cell markers (Table 3) and CD105 indicating mesenchymal stem cell (MSC) markers (Table 3) were performed via flow cytometry (Beckman Coulter, Fullerton, CA). Prior to each weekly intra-articular injection, each aliquot of AAPBSC was checked for appropriate cell counts and viability as per above (Table 3).

### 2.3. Arthroscopic Microdrilling Mesenchymal Cell Stimulation (MCS) Procedure

A single surgeon (TT) performed all arthroscopic procedures. The patients in groups 1 and 2 were scheduled for arthroscopic debridement, identification of the pathologic lesion(s) (Table 2), removal of calcified layers with preservation of the subchondral bone, creation of a stable rim of good cartilage, and multiple drillings of 2.00 mm in diameter at a depth of 4 to 6 mm. Then, immediately after the above procedure, intraoperatively and under aseptic conditions, 3 ml of AAPBSC was injected in the articular space, followed by 2 ml of GFA concentrate prepared from autologous PRP mixed with hG-CSF in group 1 but not in group 2 (Table 3). Two milliliters of HA was finally injected (Ostenil, MW = 1.2 Mdal) IA in both groups. No drain was inserted, nor was any anesthetic infiltration used. On postoperative day 7 and day 14, the second and third IA injections were given, after thawing 4.5 ml of cryopreserved AAPBSC, followed by 2 ml of GFA concentrate, freshly prepared from autologous PRP on that the same day mixed with hG-CSF in group 1 but not in group 2 (Table 3). Two milliliters of HA was finally injected (Ostenil, MW = 1.2 Mdal) IA at each occasion in both groups. Patients in group 3 only received 2 ml IA-HA (Ostenil, MW = 1.2 Mdal) at days 0, 7, and 14 without any arthroscopic surgery. Postoperatively, the patients were allowed to non-weight-bearing ambulation with axillary crutch and were discharged on the same day. All patients were monitored and telephoned on the day after their treatments. A telephone hotline was available for the patients until their next hospital visit for clinical examination. Full activity was gradually implemented over a 6-week period. Although patients were encouraged to undergo physical therapy, it was not required nor controlled.

### 2.4. Outcomes of Interest

The primary outcome measure was the need for surgical intervention at 12 months while their WOMAC score (WOMAC 3.1®; http://www.womac.org) was the secondary outcome measure and was recorded preoperatively as well as at one-, six-, and 12-month follow-up.

### 2.5. Statistical Analysis

Our sample size of sixty (60) patients could detect a 15% difference (three (3) total knee arthroplasties/replacements in control group 3 versus zero (0) in the pooled groups 1 and 2) with 90% power using a cutoff for statistical significance of 0.05. Student's *t*-test was performed to compare the means of two independent groups, and one-way analysis of variance (ANOVA) was used to compare the means of more than two independent groups while the chi-square was employed for the categorical variables. The data are presented as the mean ± standard error of the mean (SEM). A *p* value < 0.05 was considered to be significant. Statistical analysis was performed using Stata version 10.

## 3. Results

Between March 1, 2011, and April 30, 2013, sixty patients (41 females) were randomized to either of the three groups to receive treatment and all were consecutively followed up for a total of 12 months by the same observers at all follow-up points.

Patient demographics are shown in Table 1 with significantly more women with right-sided lesions enrolled. The most common cartilage lesions were seen in the medial condyle and at multiple locations. There were no statistically significant differences in the distribution of Kellgren-Lawrence and ICRS severity classification as well as in the sizes of the average lesions in the treated groups. There were no statistically significant differences in the dose numbers of autologous stem cells, in the relative percentages of the cell subsets, or in their viabilities between the treated groups (Table 3). There were no notable adverse events.

### 3.1. Primary Endpoint

The primary endpoint of surgical intervention defined as total knee arthroplasty (TKA) was necessary in 3 patients in group 3 while none in group 1 or 2 needed TKA. When groups 1 and 2 were pooled, the result reached statistical significance (Tables 4 and 5; *p* = 0.033)

### 3.2. WOMAC Score and Subscale Analysis

There were no statistically significant differences at baseline (month 0) total and subscale WOMAC scores among all 3 groups. In Table 6, all statistically significant differences within (intra) and between the three groups are displayed. Groups 1 and 2 that both were administered IA AAPBSC were also pooled (stem cell groups) and compared to group 3 (nonstem cell control group). At month 12, all groups reached statistically significant improvements within the individual (intra) groups regarding both the total score and the subscale scores.

The *total WOMAC score* was statistically significantly lower in group 1 than in group 3 and group 2 than in group 3, both at 6 and at 12 months (Tables 4 and 6). Group 1 total WOMAC score was significantly lower than that of group 2 at 6 but not at 12 months (Tables 4 and 6) indicating a quicker response in group 1. In the *pain subscale*, group 1 and group 2 scores were statistically significantly lower than those of group 3 at both 6 and 12 months. There was a statistically significant difference in the pain subscale between groups 1 and 2 already at 6 but not at 12 months (Tables 4 and 6). In the *stiffness subscale* score, only group 1 score was significantly lower than that of group 3 at 6 and at 12 months (Tables 4 and 6). Group 1 stiffness subscale score was also significantly lower than that of group 2 at 12 months. Group 2 stiffness subscale score showed a tendency to lower scores and only reached borderline significance (*p* = 0.066 and *p* = 0.053 at 6 and 12 months, resp.) (Tables 4 and 6). In the *function subscale* score, only group 1 score was significantly lower than that of group 3 at 6 and at 12 months (Tables 4 and 6). Group 1 function subscale score was also significantly lower than that of group 2 already at 6 months but not at 12 months (Tables 4 and 6). Group 2 function subscale score was statistically significantly lower than that of group 3 at 12 months (Tables 4 and 6).

## 4. Discussion

OA is characterized by the slow progressive degradation of the extracellular matrix (ECM) and the loss of the chondrogenic phenotype in articular cartilage [20]. Current therapeutic regimens address mainly pain but not degeneration and certainly not regeneration. Strategic targeting of therapeutic pathways to OA cartilage may offer potent alternatives for restoring the structure of the damaged cartilage [21]. The avascular and alymphatic nature of the articular cartilage necessitates a delivery method and a vehicle that will successfully bring in the necessary nutrients and hold the operative cells in place to exert their effect whether that is fusion with resident cells, stimulation and differentiation of local chondrocyte precursors, paracrine support, or a combination of all of the above [17, 22].

In the present study, we performed a RCT using two AAPBSC groups (one with and one without IA hG-CSF; double blinded between the groups) and compared their outcome with that of a nonstem cell group that only received HA (open between the AAPBSC groups and the nonstem cell control group). To our knowledge, this is the first report on the IA use of hG-CSF as an easily accessible, low-cost, and safe addition of a biological that offers many nonhematological advantages and has been in use for many decades in hematology [23, 24]. In the present study, the AAPBSC groups (pooled groups 1 and 2) avoided TKA at 12 months. Furthermore, IA injections of AAPBSC resulted in significant, early, and sustained (at both 6- and 12-month follow-up) total WOMAC score improvement versus the HA-only control group both in pooled and in separate comparisons. Moreover, significant differential hG-CSF effects were observed between the two AAPBSC groups (with or without IA hG-CSF addition; the only difference between the two AAPBSC groups) with an early onset (at six months) of total WOMAC score improvement, pain alleviation, and function amelioration while stiffness resolution showed a later onset at 12 months. Additionally, group 1 (with IA hG-CSF addition) showed the largest, swiftest, and most sustained improvement in all WOMAC subclasses at all evaluation points versus the HA-only control group while group 2 (without IA hG-CSF) lagged in stiffness resolution and in early function amelioration (reaching statistical significance at 12 months). All our patients were younger than 60 years of age and displayed a BMI < 27, both prognostic factors associated with increased clinical response odds [25]; however, the mean lesion size treated in our study was <7 cm^2^, larger than the 6 cm^2^ proposed cutoff seen in less favorable clinical outcomes [25]. Despite the fact that, in our study, more females than males and more right than left knees were seen in the control group, sex or side of involvement is not regarded prognostic [25].

In the current study, we have chosen to use IA AAPBSC, PRP, hG-CSF, and HA for a number of reasons. As there are many options in cells and scaffolds, the ultimate choice is always the golden means between cost, benefit, ease, and safety. Safety was the paramount in our choice. In nonhematological regenerative uses, we believe that an autologous origin of stem cells must be the only choice as the use of allogeneic sources is invariably tainted with an inherent risk of rejection and unease for potentially life-threatening complications [26, 27], the need for future immunosuppression [27], and the concern for a diminished effect due to immunovigilance [26–28] as well as worsening of inflammation in OA [27] or malignancies [29]. Moreover, allogeneic sources are usually preferred for obvious commercial and patent-related reasons and not due to ease of procurement or cost [30]. Isolated MSC of any source must invariably be expanded, and these manipulated allogeneic cells are prone to cytogenetic issues and infection/contamination risks as no uniform rules have been adopted [29–31]. We do yet not know which cell fraction(s) or specific cells are the most appropriate in regenerating cartilage. It seems that in vascular regenerative cell therapies in peripheral arterial disease, the application of whole BM or whole stimulated PBSC is more successful than methods [32] which use subfractionated cell preparations, for example, CD 133^+^ [33] or highly purified CD 34^+^ cells from PB after hG-CSF mobilization [34]. Potential feasible autologous stem cell sources for cartilage regeneration treatments include hematopoietic stem cells (HSCs) in bone marrow (BM), hG-CSF-activated/stimulated peripheral blood (PB) (the mainstay choice in stem cell collection and transplantation since many decades further ensuring patient safety [23]), and theoretically umbilical cord blood (UCB). Trabecular bone, cartilage, adipose tissue (AT), and, theoretically, umbilical cord (UC) tissues [35] also contain autologous stem cells with chondrogenic potential. The ease of procurement, collection of large numbers, and favorable costs favor PB, BM, and AT in that order. The ultimate autologous stem cell option is of course UC blood and tissues, due to ease of collection, storage, costs, safety, and availability, but there have as yet not been any autologous uses in OA employing that source [35]. The only clinical comparative study between stem cell types in cartilage regeneration is the study of Skowroński and Rutka [36] showing superior results for PBSC versus BM. The possibility of using autologous PBSCs obtained by leukapheresis after hG-CSF stimulation was first introduced by Saw et al. [13, 14, 16] who treated chondral knee lesions with subchondral drilling and five postoperative IA injections of PBSCs and HA, reporting no adverse reactions and positive histological findings. In a pilot study, Turajane et al. [18] demonstrated that a similar combination of IA AAPBSC with or without GFA along with HA in conjunction with arthroscopic MCS resulted in quality-of-life improvements measured by WOMAC and KOO scores and succeeded in regenerating articular cartilage in early osteoarthritic knee disease that failed conservative treatment versus noncellular interventions without adverse reactions. Subsequently, in their assessment of the chondrogenic differentiation potential of AAPBSC on human early osteoarthritic cancellous tibial bone scaffolds seeded with hG-CSF-mobilized PB-derived stem cells for cartilage regeneration, temporally and sequentially increased expression of cartilage-relevant genes such as Sox9, collagen type II (COL-2), and aggrecan (AGGR) as well as histological evidence of increased and incorporated proteoglycan and glycosaminoglycan contents was demonstrated [17]. A later RCT, also by the group of Saw et al. [14], similarly documented (via MRI and histological evaluations) positive clinical outcomes at 24 months using autologous PBSC, versus HA controls.

Mesenchymal stromal cells (MSC) and mesenchymal progenitor cells (MPC) as well as endothelial progenitor cells (EPC) are considered to be chondrogenic [37]. All those types of stem cells together and in great numbers have been found in PB, BM, and UC only [37]. It has previously been reported [17] that AAPBSC displayed positive staining for the mesenchymal surface markers CD29, CD44, CD90, and CD105 and exhibited especially high levels of expression of CD29 and CD44, which stained more than 80% of the total cell population. In the present study, our administered cell preparations contained a sufficient and viable mixture of MSC and EPC (CD105 and CD34) to initiate resurfacing. EPC from both the CD34^+^ and the CD105^+^ populations may be of importance in ensuring an adequate balance between availability and handling of oxygen in developing growth of cartilage to preserve chondrocyte survival and may also act together with vascular endothelial growth factor (VEGF) in ensuring vascular supply [38]. In the present study, we chose to harness the regenerative potential of *autologous PRP* prepared on the days of the IA injections, hypothesizing that the release of growth factors that occurs with platelet rupture may deliver a crucial secretome of transforming growth factor (TGF) beta, insulin-like growth factor 1 (IGF-1), fibroblast growth factor (FGF), VEGF, bone morphogenetic proteins (BMP), and platelet-derived growth factor (PDGF), all known to promote stimulation of blood vessel, chondrocyte, and ECM formation [39–41]. Chondrogenic markers (Sox9, AGGR, and COL-2) have been confirmed to increase following PRP administration suggesting that TGF beta in PRP may enhance MSC proliferation and cause chondrogenic differentiation of MSC in vitro [42]. CD105 is a TGF beta receptor that may play a role in mediating interactions between HSC and MSC in the marrow [43]. Another possible role for CD105 on MSCs may be in mediating TGF beta signaling during chondrogenic differentiation. All TGF beta isoforms [44] are capable of inducing MSCs from human and other species along the chondrogenic pathway. Moreover, TGF beta in PRP works along with FGF2 to assist in the migration of stromal cells to the site of injury [45]. The *HA carrier* we have used, FGF2 in our PRP and the MSC in the AAPBSC, may further interact through the abundant CD44 in our preparation [17] to facilitate MSC migration and adherence to a chondral defect [45]. The *addition of IA hG-CSF* may further enhance cartilage regeneration by attracting additional bone marrow stem cells to home in, aiding angiogenesis in a conducive environment, regulating the proliferation, and enhancing the migration or differentiation of the adult MSCs [46] locally. Moreover, the advantageous effects of hG-CSF on bone tendon integration may extend to the subchondral area [46]. Interestingly, Fujihara et al. identified G-CSF as an inducer of FasL on chondrocytes, and G-CSF-treated tissue-engineered cartilage showed less infiltration of macrophages, with increased formation of cartilage after transplantation. [47], and this observation may offer an additional explanation to the effects observed in our model [17]. Furthermore, hG-CSF has recently been shown to attenuate the impact of aging on BM stem cells and recover age-related functional decline by significantly improving their proliferation activity and growth factor production such as nerve growth factor (NGF) and brain-derived neurotrophic factor (BDNF). Both of the latter are found in the synovial membrane, expressed in articular chondrocytes, and play a role in knee arthritis [48], thus bypassing age-related limitations and objections in using AAPBSC in cartilage repair. hG-CSF is not only a hematopoietic cytokine with bone marrow stem cell mobilizing properties [49]. Encouraging nonhematological effects and target organs have been identified in recent years [50–53]. hG-CSF and its respective receptor are expressed in the human central nervous system (CNS) and may be an important part of the brain's endogenous system of protection [50, 52]. Its neuroprotective and neuroregenerative potential as well as its antiapoptotic and anti-inflammatory properties has been acknowledged and demonstrated in human studies [50–53]. Early systemic hG-CSF treatment attenuates neuropathic pain after peripheral nerve injury through activation of mu opioid receptors on the injured nerve [54], induces swift pain relief in diabetic foot gangrene in humans [55], and promotes a dramatic therapeutic effect on a rodent model of diabetic neuropathy, which was attributed, at least in part, to the actions of bone marrow-derived cells [56]. In concordance to the above results, early and sustained relief in pain was a significant finding in the present study followed by improvements in function as the latter may be compromised by excess pain. The effects of G-CSF on arthritic pain may be conflicting [57], but in some situations, G-CSF has even been considered to be an anti-inflammatory immunomodulator where the effects of systemic and/or local pharmacological exogenous doses of hG-CSF might differ from those of endogenous G-CSF [58]. Exogenous hG-CSF mobilizes hematopoietic cells from the bone marrow which, in turn, probably changes the composition of peripheral blood cell populations to less mature and perhaps less inflammatory phenotypes, thereby changing the nature of the dynamic cell populations available to migrate into the site of inflammation [58]. Moreover, hG-CSF appears crucial for skeletal myocyte development and regeneration [59] and positively affects the satellite cell population aiding the preservation of the satellite stem cell pool [60] which in turn supports long-term muscle regeneration and functional maintenance [60], all factors that may be behind improvements in function and stiffness observed in the present study.

All above findings justify our choices of HA, PRP, hG-CSF, and AAPBSC, together orchestrating a powerful regenerative concert resulting in the composition of hyaline cartilage. HA and/or PRP in isolation have not been associated with disease modification and structural changes [45].

As mentioned earlier, AT may contain autologous MSC with chondrogenic potential in its stromal vascular fraction (SVF), but the prospect of using stem cells from this heterogeneous cell population containing preadipocytes and immune cells along with their secretome of inflammatory immunokines from a widely active endocrine tissue implicated in the pathogenesis and sustainment of cardiovascular and metabolic diseases seems absurd [61, 62]. Furthermore, a crucial and positive relationship between obesity and cancer is well known, and the purported immunomodulating and angiogenic properties of AT MSC raise suspicions about a possible causation [59, 63]. Moreover, ex vivo expansion and long-term cultures with increased numbers of passages are associated with extensive morphological and functional changes of MSCs [31] and subsequently the concern that these cells may accumulate stochastic mutations which may lead to the risk of malignant transformation [31, 61].

Evidence is accumulating [17, 37] that the primary benefit of the cellular components appears to derive from their paracrine effects on the native tissue through immunomodulation and/or gene up-/downregulation [17] rather than themselves providing the building blocks for regeneration, and therefore, it would be unwise to solely ascribe the resulting chondrogenesis to MSC. The cellular doses administered in the present study were adequately high at all 3 occasions in both groups that received stem cells. Dose response analyses have failed to assign better outcomes with higher doses [3], and in this sense, repeated MSC cultures to ensure higher cell doses seem to be an unjustified risk at a great cost [31]. When autologous PB or BM is the source of the cellular therapy, synergistic action of all cellular components and their compatible and synchronous secretome seems to confer all the necessary endogenous advantages. Allowing native MSC and EPC contained in the autologous HSC fraction to cross-talk with local tissues may offer potential advantages in the treatment of OA [64]. HSCs have also been hypothesized to be the source of all cells in our body which makes PBSC the ideal cellular source both in terms of easiness of collection, cell numbers, safety, and efficacy [65]. The downsides of MSC expansion with cytogenetic instability and risk of malignant transformation are thus avoided [31] as well as the ever-present risk of immune rejection when allogeneic MSCs (especially when chondrogenic differentiation is induced) are injected [27]. Allogeneic, cultured, and expanded stem cells may present wider and more challenging issues than the well-known onerous immunological tissue antigen MHC incompatibility [27]. Epigenetic dysynchronicity with incongruent gene methylation status and thus discordant gene products, divergent secretomes, and microRNA products as well as differences in nutrient sensing between donor and recipient tissues are issues that are only recently being researched and may present even bigger regenerative hurdles [66].

## 5. Conclusions

In this RCT, we have shown avoidance of TKA in the AAPBSC groups at 12 months and potent, early, and sustained symptom alleviation through the GFA (PRP and hG-CSF) versus HA alone. Differential effects of hG-CSF were noted with an earlier onset of symptom alleviation throughout. In a clinical setting, harnessing the human body's ingenuity to merge safe and effective components seems intuitive, and thus, the intra-articular coadministration of HA, PRP, hG-CSF, and AAPBSC appears as the golden means to alleviate pain and disability in a progressive condition in an aging population where no disease-modifying pharmacological therapeutic alternatives exist. Autologous PBSC-based cell therapies offer a safe and exciting possibility in the treatment of OA and importantly show promise in disease modification, with potential inhibition of progression and, in the present study, evidence of reversal of this degenerative process. Further randomized controlled trials are needed to evaluate this treatment modality in osteoarthritis management.

## Figures and Tables

**Table 1:** Patient demographics.

	Group 1	Group 2	Group 3	*p* value
Number (patients)	20	20	20	1.00
Male	10	3	6	0.005
Female	10	17	14	0.005
Number (knees)	20	20	20	1.00
Left knee	10	9	6	0.042
Right knee	10	11	14	0.042
Age (yr), mean (SD)	54.9 (±6.1)	55.4 (±2.3)	54.7 (±3.5)	0.468
Bodyweight (kg), mean (SD)	70.7 (±14.0)	66.2 (±12.7)	65.9 (±8.7)	0.245
Height (cm), mean (SD)	164.5 (±7.4)	159.8 (±8.4)	158.1 (±5.7)	0.005
BMI, mean (SD)	25.9 (±3.9)	25.9 (±4.1)	26.2 (±3.0)	0.789
TF (SD)	1.9 (±0.8)	1.95 (±0.9)	1.9 (±0.8)	0.834

**Table 2:** Cartilage pathology (Gr = group; MC = medial condyle; LC = lateral condyle; PF = patellar facies; MUL = multiple locations; N/A = not applicable/available as Gr 3 did not receive any arthroscopic surgery).

	Group 1	Group 2	Group 3
Number (patients)	20	20	20
Kellgren-Lawrence classification Gr 2-3	Gr 2 = 11	Gr 2 = 11	Gr 2 = 11
	Gr 3 = 9	Gr 3 = 9	Gr 3 = 9
Intraoperative location	MC = 9	MC = 8	N/A
	LC = 1	LC = 1	
	PF = 3	PF = 3	
	MUL = 7	MUL = 8	
ICRS classification	III = 12, IV = 8	III = 10, IV = 10	N/A
Size of lesion (cm)	2.5 × 2.7	2.5 × 2.6	N/A

**Table 3:** Activated autologous peripheral blood stem cell collection counts (total nucleated cells (TNC), CD34^+^ cells, and CD105^+^ cells) and cell viability at different times of injection in all patients.

Group	Day	TNC × 10^3^/l in 3 ml	CD34 as % of TNC		CD105 as % of TNC		Viability (%)	
			Fresh	Frozen	Fresh	Frozen	Fresh	Frozen
Group 1	0	1095	0.47		0.83		96	
	7	1264		0.81		1.28		78.9
	14	1276		1.17		1.62		80.72
Group 2	0	1143	0.44				90.56	
	7	1252		0.78		1.19		81.0
	14	1253		1.13		1.5		81.4
Group 3	0	^∗^	^∗^	^∗^	^∗^	^∗^	^∗^	^∗^
	7	^∗^	^∗^	^∗^	^∗^	^∗^	^∗^	^∗^
	14	^∗^	^∗^	^∗^	^∗^	^∗^	^∗^	^∗^

^∗^Not applicable.

**Table 4:** 

Time/groups	6 months				12 months				
WOMAC score	Total	Pain	Stiffness	Function	Total	Pain	Stiffness	Function	Total knee arthroplasty (TKA)
Group 1 versus group 2	S	S	NS	S	NS	NS	S	NS	
Group 1 versus group 3	S	S	S	S	S	S	S	S	
Group 2 versus group 3	S	S	BS	NS	S	S	BS	S	
Groups 1 + 2 versus group 3					S				S

S = significant; NS = nonsignificant; BS = borderline significance. Group 1 improved significantly quicker than group 2 in the total WOMAC score at 6 months, but that difference disappeared at 12-month follow-up. Similarly, pain and function improved quicker in group 1 than in group 2 at 6 months, but no difference was noted at 12 months while stiffness was better in group 1 at 12 but not at 6 months. Group 1 scores (stem cell and G-CSF group) were superior to those of group 3 (nonstem cell control group) at all points and in all modalities, total or subscale. Group 2 improved significantly versus the control group 3 at 6 and 12 months on total WOMAC score and pain similar to group 1 versus group 3, but stiffness improvement was only of borderline significance at both time points while function improved significantly only at 12 months. Pain subsided quickest in group 1 versus all groups at 6-month follow up and may have led to increased function as well since patients become pain free. Stiffness depends more on connective tissue rearrangements as well as on new cartilage which probably needs time. The stem cell group 1 with G-CSF displayed a quicker response than the control group 3 at all time points and also than the non-G-CSF stem cell group 2 at 6 months (except for stiffness).

**Table 5:** Avoidance of total knee arthroplasty (TKA) in pooled activated autologous peripheral blood stem cell (AAPBSC) groups versus the nonstem cell group.

12-month follow-up	Group 1 (*N* = 20)	Group 2 (*N* = 20)	Group 3 (*N* = 20)	*p* value
Surgical intervention (Total knee arthroplasty (TKA))	None (0)	None (0)	Three (3)	Pooled groups 1 and 2 versus group 3 at 12 months: *p* < 0.033

**Table 6:** 

WOMAC score	Gr 1 (*N* = 20)				Gr 2 (*N* = 20)				Gr 3 (*N* = 20)			
Time (months)	0	1	6	12	0	1	6	12	0	1	6	12
Total	218.5	131.1	68.7^bb,cc,dd^	52^a,b^	212.2	154.3	114.2^cc,ee^	75^c,d^	215.3	170.2	134.6^dd,ee^	126.8^a,c,e,aa,bb^
Pain	95.5	60	30.5^ff,gg^	28^f,g^	93.5	69	50^ff^	30^g,h^	96	83	64^gg^	57^k,f,h,j^
Stiffness	51	27.5	15.5^hh^	9^l,m,n^	49.5	32.5	21.5^ii^	20^o,l^	50	32.5	26.5^hh,ii^	31.5^q,r,l^
Function	69.3	43.6	22.7^jj,kk^	15^s,t^	69.3	52.8	42.7^jj^	25^x,y^	69.3	54.7	45.1^kk^	38.8^z,s,x,za^

Total: a = Gr 1 versus Gr 3, *p* < 0.001; b = intra Gr 1 month 0 versus 12, *p* < 0.0001; c = Gr 2 versus Gr 3, *p* < 0.001; d = intra Gr 2 month 0 versus 12, *p* < 0.0001; e = intra Gr 3 month 0 versus 12, *p* = 0.001; aa = pooled Gr 1 & 2 versus Gr 3, *p* < 0.001; bb = Gr1 month 6 versus Gr3 month 12, *p* < 0.001; cc = Gr 1 month 6 versus Gr 2 month 6, *p* = 0.004; dd = Gr 1 month 6 versus Gr 3 month 6, *p* = 0.001; ee = Gr 2 month 6 versus Gr 3 month 6, *p* = 0.064. Pain: f = Gr 1 versus Gr 3, *p* = 0.003; g = intra Gr 1 month 0 versus 12, *p* < 0.0001; h = Gr 2 versus Gr 3, *p* = 0.003; i = intra Gr 2 month 0 versus 12, *p* < 0.0001; j = pooled Gr 1 & 2 versus Gr 3, *p* = 0.004; k = intra Gr 3 month 0 versus 12, *p* = 0.001; ff = Gr 1 pain month 6 versus Gr 2 month 6, *p* = 0.012; gg = Gr 1 pain month 6 versus Gr 3 month 6, *p* = 0.002. Stiffness: l = Gr 1 versus Gr 2, *p* = 0.0001; m = Gr 1 versus Gr 3, *p* = 0.0001; n = intra Gr 1 month 0 versus 12, *p* < 0.0001; o = intra Gr 2 month 0 versus 12, *p* < 0.0001; q = pooled Gr 1 & 2 versus Gr 3, *p* = 0.0001; r = intra Gr 3 month 0 versus 12 *p* = 0.001; Gr 2 versus Gr 3, *p* = 0.053; hh = Gr 1 stiffness month 6 versus Gr 3 month 6, *p* = 0.027; ii = Gr 2 stiffness month 6 versus Gr 3 month 6, *p* = 0.066. Function: s = Gr 1 versus Gr 3, *p* = 0.001; t = intra Gr 1 month 0 versus 12, *p* < 0.0001; x = Gr 2 versus Gr 3, *p* = 0.003; y = intra Gr 2 month 0 versus 12, *p* < 0.0001; z = pooled Gr 1 & 2 versus Gr 3, *p* = 0.001; za = intra Gr 3 month 0 versus 12, *p* = 0.0001; jj = Gr 1 function month 6 versus Gr 2 month 6, *p* = 0.011; kk = Gr 1 function month 6 versus Gr 3 month 6, *p* = 0.006.
